# HIV-associated neurocognitive disorders in sub-Saharan Africa: a pilot study in Cameroon

**DOI:** 10.1186/1471-2377-10-60

**Published:** 2010-07-13

**Authors:** Georgette D Kanmogne, Callixte T Kuate, Lucette A Cysique, Julius Y Fonsah, Sabine Eta, Roland Doh, Dora M Njamnshi, Emilienne Nchindap, Donald R Franklin, Ronald J Ellis, John A McCutchan, Fidele Binam, Dora Mbanya, Robert K Heaton, Alfred K Njamnshi

**Affiliations:** 1 Department of Pharmacology and Experimental Neurosciences, University of Nebraska Medical Center, Omaha, NE, USA; 2 Faculty of Medicine and Biomedical Sciences, University of Yaoundé I, Yaoundé, Cameroon; 3 Department of Neurology, Yaoundé Central Hospital, Yaoundé, Cameroon; 4 HIV Neurobehavioral Research Center, University of California, San Diego, USA; 5 Brain Sciences, University of New South Wales, Sydney, Australia; 6 HIV Day-Care Hospital, Yaoundé Central Hospital, Yaoundé, Cameroon; 7 University Hospital Center, Yaoundé, Cameroon

## Abstract

**Background:**

The disease burden of human immunodeficiency virus (HIV) - acquired immunodeficiency syndrome (AIDS) is highest in sub-Saharan Africa but there are few studies on the associated neurocognitive disorders in this region. The objectives of this study were to determine whether Western neuropsychological (NP) methods are appropriate for use in Cameroon, and to evaluate cognitive function in a sample of HIV-infected adults.

**Methods:**

We used a battery of 19 NP measures in a cross-sectional study with 44 HIV+ adults and 44 demographically matched HIV- controls, to explore the validity of these NP measures in Cameroon, and evaluate the effect of viral infection on seven cognitive ability domains.

**Results:**

In this pilot study, the global mean z-score on the NP battery showed worse overall cognition in the HIV+ individuals. Significantly lower performance was seen in the HIV+ sample on tests of executive function, speed of information processing, working memory, and psychomotor speed. HIV+ participants with AIDS performed worse than those with less advanced HIV disease.

**Conclusions:**

Similar to findings in Western cohorts, our results in Cameroon suggest that HIV infection, particularly in advanced stages, is associated with worse performance on standardized, Western neurocognitive tests. The tests used here appear to be promising for studying NeuroAIDS in sub-Saharan Africa.

## Background

Neurological complications affecting the central and peripheral nervous systems are common in HIV infection. The vast majority of studies on HIV-related neurocognitive disorders were performed in developed countries, on patients infected with HIV-1 subtype B [for recent reviews, see [1-4]]. Two-thirds of the estimated 33.2 million individuals currently living with the human immunodeficiency virus (HIV)/acquired immunodeficiency syndrome (AIDS) worldwide are in sub-Saharan Africa (SSA) and are infected with mostly non-B HIV subtypes. Thus, it is important to diagnose and categorize HIV effects on the central nervous system (CNS) in SSA [4,5].

Autopsy studies of HIV-infected individuals have shown that the brain is the second organ most frequently affected by HIV infection [6]. Studies of neuroAIDS pathogenesis have established that following HIV infection, the virus rapidly crosses the blood-brain barrier and enters the brain, where it productively infects brain macrophages and microglia [for recent reviews, see [3,7-9]]. Toxic HIV proteins and cytokines secreted by infected mononuclear phagocytes in the brain cause dysfunction of other CNS cells and neuronal death [10-12]. This HIV-induced brain pathology often results in behavioral, motor and cognitive abnormalities referred to as HIV-associated neurocognitive disorders (HAND) [13]. HIV-associated dementia (the most severe form of HAND) has been described as a sub-cortical dementia, yet clinical and pathological studies have also shown an association between cortical neurodegeneration and HIV-associated neurocognitive impairment in infected patients [4,14,15].

Like many countries in SSA, Cameroon still has a heavy burden of the HIV/AIDS epidemic. According to recent estimates, the prevalence of HIV infection in the overall adult population of Cameroon is 5.1%, with prevalence of 8.3% in large metropolitan areas like the capital city Yaoundé, 7.4% among women, and 26.4% in female sex workers [16-18]. Furthermore, Cameroon has a high genetic diversity of HIV strains including several group M HIV-1 subtypes, HIV-1 groups O and N, circulating recombinant forms and unique recombinant forms [19-23]. Therefore, it is important to understand the neurological and neurocognitive effects of HIV infection in this country.

A comprehensive NP battery was recently adapted and successfully used to produce normative data in healthy children in Cameroon [24]. Also, we have screened HIV-infected adults in Yaoundé for HAND and its risk factors using the International HIV Dementia Scale (IHDS) [25,26]. Although the IHDS is simple and rapid, it is neither comprehensive nor able to assess specific cognitive functional domains. The goal of the current study was to adapt the HIV Neurobehavioral Research Center (HNRC, University of California at San Diego) international NP test battery in Cameroon, and to explore its validity for the detection of HAND in infected Cameroonians. This battery included 19 test measures designed to evaluate several ability domains known to be vulnerable to HIV effects on the brain, including abstraction/executive function, speed of information processing, attention and working memory, verbal fluency, motor ability, learning and delayed recall [27-29]. This preliminary report describes the first attempt to examine the effect of HIV infection on specific cognitive domain functions in infected individuals in Cameroon.

## Methods

### Setting and study design

This study is part of an ongoing project funded by the United States National Institute of Mental Health aimed at studying the neurological complications of HIV infection in Cameroon. The study was approved by the Cameroon National Ethics Committee, as well as the Institutional Review Board of the University of Nebraska Medical Center, and the Cameroon Ministry of Public Health delivered the administrative authorization for this research. This was a cross-sectional study comparing HIV-seropositive (HIV+) cases and HIV-seronegative (HIV-) controls. The HIV+ subjects were further classified as AIDS and non-AIDS based on clinical and immunological criteria: Center for Disease Control (CDC) clinical disease stage and CD4 T-lymphocyte counts [30,31]. All clinical and neuropsychological testing was performed in the Neurology Department of the Yaoundé Central Hospital, Yaoundé, Cameroon.

### Training and adaptation of the neuropsychological battery

For this study, we used the HNRC international NP test battery, a battery that has been shown to be sensitive in detecting HAND in the USA, India, and China [27-29,32]. This battery (Table 1) was administered by psychologists trained in psychometrics. To adapt NP and neuromedical procedures to Cameroon settings and ensure standardization, the Cameroon investigators (GDK, AKN, and CK) were trained and certified by American neuropsychologists and psychometrists (RKH, LAC, DRF) at the HNRC, University of California San Diego, U.S. The Cameroon investigators trained at HNRC then trained other Cameroonian examiners (psychologists, RD, SE) in Yaoundé, Cameroon who were certified by HNRC following review of their video-recorded testing sessions using staff volunteers from the Neurology Department, Yaoundé Central Hospital, Cameroon. We translated all tests in the battery and test instructions into French. For the Hopkins Verbal Learning Test-Revised (HVLT-R), we used the version previously translated into French and validated by Rieu and colleagues [33]. The French version of the HNRC NP battery was tested, piloted, and approved by all bilingual (French- and English-speaking) scientists on the team (GDK, CTK, AKN, LAC). Selected NP tests were back-translated into English and back-translated tests were similar to the original English version of the tests. At the beginning of the study in Cameroon, quality assurance reviews were conducted by HNRC scientists on test forms of the first 5 visits, and thereafter on randomly selected 5 to 10% of all visits.

**Table 1:** Neuropsychological (NP) tests battery and Z-scores (mean ± SD) in the HIV+ group

Ability domain	NP tests (reference)	HIV+ (N = 44)	*P *<	*d*
**Verbal**	Letter Fluency [34,35]	-0.07 ± 1.02	0.72	.07
	Animal Fluency [35]	-0.17 ± 0.82	0.37	.18
	Action Fluency [36,37]	-0.15 ± 0.70	0.42	.17

**Executive Functions**	Category Test [38]	-0.53 ± 0.95	**0.01**	**.52**
	WCST-64 Total Errors [39]	-0.88 ± 1.40	**0.002**	**.68**
	Color Trails II [40]	-0.34 ± 1.07	0.34	.31

**Speed of information processing**	TMT-A (U.S. War Department, 1944)	-0.04 ± 0.84	0.83	.04
	WAIS-III Digit symbol [41]	-0.33 ± 0.86	**0.09**	.34
	WAIS-III Symbol Search [41]	-0.33 ± 0.83	0.10	.34
	Stroop Color [42]	-0.30 ± 0.97	0.15	.29
	Color Trails I [40]	-0.17 ± 0.95	0.41	.17

**Memory Learning**	BVMT-R Learning [43]	-0.33 ± 1.14	0.15	.29
	HVLT-R Learning [44]	-0.08 ± 1.20	0.71	.07

**Memory Recall**	BVMT-R Delay Recall [43]	-0.42 ± 1.30	**0.09**	.35
	HVLT-R Delay Recall [44]	-0.18 ± 1.05	0.41	.17

**Working Memory**	PASAT 50 [45,46]	-0.49 ± 0.96	**0.02**	**.48**
	WMS-III Spatial Span [47]	-0.41 ± 0.89	**0.04**	**.41**

**Motor function**	Grooved Pegboard DH [48]	-0.18 ± 1.17	0.42	.16
	Grooved Pegboard NDH [48]	-0.26 ± 1.23	0.28	.22

**Mean Z-score**		-0.30 ± 0.64	**0.05**	.**41**

Raw scores were transformed into z-scores using the control's mean and standard deviation. P represents statistical significance when comparing mean z-scores or HIV+ and mean z-scores of HIV- group. **Abbreviations**: SD: standard deviation; WCST: Wisconsin Card Sorting Test; TMT-A: Trail Making Test part A; WAIS: Wechsler Adult Intelligence Scale; BVMT-R: Brief Visuospatial Memory Test-Revised; HVLT-R: Hopkins Verbal Learning Test-Revised; PASAT: Paced Auditory Serial Addition Test; WMS: Wechsler Memory Scale; DH: dominant hand; NDH: non-dominant hand.

### Neuropsychological tests

Table 1 show the tests used in this study for a comprehensive neuropsychological assessment of subjects, grouped by ability domain:

*Verbal fluency *tests included tests of verbal, animal and action fluency, which measure the ability of generating words beginning with a given letter [34,35] or by category [36,37].

*Executive functions *tests included the Halstead Category Test [38], Wisconsin Card Sorting Test-64 (WCST-64) [39], and Colors Trails II [40]. The WCST-64 is a measure of frontal lobe function that assesses the ability to learn concepts, perseverance and competence in abstract reasoning. The Category test is also a measure of frontal lobe function and includes 7 subtests: subtests I and II evaluate number counting and attention; subtests III and VI measure visual abstract reasoning and memory respectively; subtests IV and V measure visual perception and spatial orientation respectively, while subtest VII evaluates learning and retention of the concepts associated with other subtests [38].

*Speed of information processing *tests: the Wechsler Adult Intelligence Scale (WAIS)-III Digit Symbol and WAIS-III Symbol Search tests [41] produces measures of processing speed, visual perception, attention, concentration, visual-motor coordination, motor and mental speed. The Stroop Color and Word tests [42] measures cognitive processing and can provide valuable diagnostic information on brain dysfunction, cognition, mental speed and mental control. The Color Trails and Trail Making Test produces measures of attention, visual searching, mental processing speed, and measure the ability to mentally control simultaneous stimulus pattern [40].

*Memory learning and memory recall *tests: the Brief Visuospatial Memory Test-Revised (BVMT-R) [43] measures visual learning and memory while the HVLT-R [44] assesses verbal learning and memory. Both BVMT-R and HVLT-R also assess recognition and recall.

*Working memory *tests: the Paced Auditory Serial Addition Test (PASAT)-50 is a measure of cognitive function that specifically assesses the processing speed of auditory information, concentration, flexibility, mental calculation and mental tracking abilities [45,46]. The Wechsler Memory Scale (WMS) Spatial Span provides an estimate of general memory functioning and is sensitive to memory impairments associated with various clinical conditions [47].

*Motor function *test: the Grooved Pegboard test (dominant and non-dominant hand) measures performance speed and requires complex visual-motor coordination [48].

### Participants and data collection

HIV+ individuals and seronegative controls were recruited from January 2008 to June 2009 at 1) the HIV voluntary counseling and testing sections of the Daycare Hospital in the Yaoundé Central Hospital; and 2) the Health and Social Welfare Centre of the University of Yaoundé I. The purpose of the study and research procedures were fully explained to participants and adults 18 to 60 years old who gave a written consent were allowed to participate in the study. The exclusion criteria were: 1) present or past history of CNS disease unrelated to HIV, 2) head trauma, 3) current alcohol intoxication (blood alcohol content of each participant was measured using a Breathalyzer), 4) known psychiatric disease or treatment with antipsychotic drugs, and 5) ongoing systemic illness or fever with temperature of 37.5°C or higher. All subjects enrolled spoke French as their primary language and interviews were conducted in French. All participants provided demographic information, underwent a thorough neurological assessment and a complete medical history and physical examination by neurologists at Yaoundé Central Hospital, before NP testing, to detect any focal neurological deficit suggestive of CNS opportunistic infection. This thorough clinical assessment of each subject combined with review of his or her prior medical history and laboratory data, ensured that potential confounding factors such as CNS opportunistic infections were ruled out.

Beck Depression Inventory [49] and NP tests were administered to each subject by trained psychologists/psychometrists in the Neuropsychology Room of the Neurology Department: a private, quiet and well-lit room. Following neurological and general medical assessment and NP testing, blood samples were collected for serology. The HIV status of each participant was determined using the rapid immunochromatographic HIV-1/2 test (Abbott Diagnostics, Chicago, IL, USA) and the Murex HIV antigen/antibody Combination ELISA (Abbott Diagnostics). A participant was considered HIV+ if they tested positive for the 2 tests and HIV- if negative for both tests, and discordant if positive for only one test. No discordant result was observed in this study. The CD4 T-lymphocyte count of infected individuals was determined by flow cytometry, using a Fluorescence Activated Cell Sorting (FACS) Count Instrumentation System (BD Biosciences, San Jose, CA, USA). For viral load quantification we used the Abbott Real-Time Viral Load Test, with Abbott m2000 system (Abbott Diagnostics). All biological tests were done in the Haematology Laboratory of the University Hospital Center, Yaoundé, Cameroon.

### Data analysis

Eighty-eight (88) individuals, of whom 44 were HIV+ and 44 HIV- completed the NP battery. Both groups were well matched for age, education, and gender (Table 2). Raw scores were transformed into z-scores using the control group's mean and standard deviation. All z-scores were coded so that increasingly negative scores indicated worse performance. The t-test was used to statistically compare the HIV+ and HIV- groups on each of the 19 NP measures, and Cohen's *d *effect sizes were also computed. A summary score on the NP battery was obtained by averaging the 19 individual z-scores to reflect overall level of NP performance, and scores on measures in each of the seven ability domains were averaged to create domain summary scores. We also explored the magnitude of demographic effects on test performance in our Cameroon participants, which would need to be controlled in a norms development project before the tests could be used confidently to classify cognitive impairment in individual Cameroonians. Thus, the individual effects of age, education and gender were tested using standard regression analyses, separately in the HIV- and HIV+ groups, and for each cognitive ability domain. All effect sizes were corrected for small samples size bias using Hedges' correction. The seronegative control group, the non-AIDS and AIDS subgroups were compared using analysis of variance (ANOVA) and post hoc Dunnett's comparisons on the overall mean z-score and mean z-scores on the seven ability domains. Secondary analyses included as covariates those demographic variables that were significant in univariate analyses. Analyses were conducted using the JMP statistical software version 7.0 (SAS Inc; 2008).

**Table 2:** Demographic and clinical characteristics of the HIV+ and HIV- groups

	HIV-	HIV+	*P value*
	(N = 43)	(N = 44)	
Age	33.26 ± 11.40	34.91 ± 10.25	.48

Education	12.60 ± 4.44	12.11 ± 3.9	.58

Speak French	100%	100%	

Sex (% female)	58.1%	61.4%	.76

AIDS %	-	50%	

BDI-II	14.64 ± 9.48	13.48 ± 8.88	.56

BDI-II: Beck depression inventory 2^nd ^edition [49].

## Results

### Demographic, clinical and laboratory characteristics

A total of 88 (44 HIV+ and 44 HIV-) adult Cameroonians were evaluated. Participants' demographic, clinical and laboratory characteristics are summarized in Table 2. All participants were screened for sub-optimal effort using the Hiscock forced-choice recognition test cut-off of less than 90%. Only one individual in the control group scored in the sub-optimal effort range and was excluded from further analyses. There was no statistically significant difference between cases and controls in age, education, or sex (% female) (Table 2). Of the 44 HIV+ individuals, 22 (50%) were classified as non-AIDS and 22 as having AIDS (Table 3): CD4 T-lymphocytes counts below 200 cells/μl and AIDS indicator conditions as specified in the CDC AIDS case definition criteria [30,31]. Fifteen of the 44 HIV+ individuals (34%) were on antiretroviral therapy (ART) and as expected their viral load was significantly lower than that of AIDS and non-AIDS HIV+ individuals who were not on ART (Table 3).

**Table 3:** Laboratory characteristics of the HIV+ group

	Mean CD4 ± SE	*P*	Mean viral load ± SE	*P*
	(Cells/μl) [IQR]		(Log copies/ml) [IQR]	
**HIV+ Non-AIDS **(N = 22)	496 ± 51.5		2.86 ± 0.47	
	[597.5 - 323]		[4.7 - 0.65]	
		0.0005		0.1
			
**HIV+AIDS **(N = 22)	251 ± 38.9		4 ± 0.5	
	[360.5 - 117]		[5.88 - 1.85]	

**HIV+ Non-AIDS**	493.4 ± 70.5		3.95 ± 0.38	0.0001
**No ART **(N = 16)	[620 - 268]		[5 - 2.78]	
		NS		
			
**HIV+ Non-AIDS**	502.8 ± 47.6		0.33 ± 0.3	
**ART **(N = 6)	[620.5 - 375]		[0.98 - 0.0]	

**HIV+AIDS**	225.5 ± 42		5.12 ± 0.46	
**No ART **(N = 13)	332.5 - 128]	NS	[6.1 - 4.84]	0.0014
			
**HIV+AIDS**	292.5 ± 77.5		2.15 ± 0.67	
**ART **(N = 9)	[471.5 - 109]		[3.65 - 0.65]	

SE: standard error; IQR: interquartile range; ART: Antiretroviral therapy; N: sample size; NS: not significant.

### NP tests performance of controls and HIV-infected patients in Cameroon

Table 1 contains the z-scores of the HIV+ and HIV- groups for all 19 test instruments used in this study. The summary mean z-scores across all 19 test measures showed that HIV+ group performed significantly less well in NP testing than HIV- controls (P < 0.05). Comparisons of individual z-scores for each of the 19 test measures showed that for the WCST-64, PASAT-50, Category test, and Spatial Span, the NP performance of HIV+ individuals was significantly lower than that of HIV- controls. Computation of effect sizes further showed that effect sizes were medium for the Category Test and WCST-64 (both measures of executive function), and small-to-medium for PASAT-50 and Spatial Span (measures of working memory), although not statistically significant with the current sample sizes. Small-to-medium HIV status effects sizes were also noted on another measure of executive function (Color Trails-II), as well as on measures of speed of information processing (Digit Symbol, Symbol Search) and visual episodic memory (BVMT-R). Finally, small effect sizes for HIV status were also seen for another measure of processing speed (Stroop Color Naming) and a measure of motor function (Grooved Pegboard, Non-dominant Hand). Although mean scores on all tests in the battery were worse for the HIV+ group than the HIV- group, only a minimal effect (*d *< 0.2) was observed on the remaining eight measures in Table 1 (especially on tests of verbal fluency and verbal episodic memory).

As expected, very substantial demographic effects were present in the data and influenced NP performance. Age and education independently and in combination predicted global mean z-scores and ability domain mean z-scores in both HIV- and HIV+ groups, with demographic effects more pronounced in the control group (Table 4). Older age and lower education were associated with lower NP performance; gender had no effect on NP performance, except for verbal tests where gender significantly influenced mean z-scores in the control group (Table 4).

**Table 4:** Individual (Pearson r) effects of age, education and sex on global (mean z-score) and ability domains (mean ability z-score) neuropsychological performance

	HIV-	HIV+
	**Age Education Sex**	**Age Education Sex**

**Mean Z-score**	-.57 ***;.72****; -.01	-.38 **;.55***; .14

**Verbal**	-.41 **; .53**; -.38*	-.005;.47**; .10

**Executive function**	-.50 ***;.55***; -.18	-.22;.38*; .00

**Speed information processing**	-.52 ***;.72****; -.03	-.33*;.47**; .08

**Memory Learning**	-.42 ***;.49****; -.11	-.43 **;.48***; .17

**Memory Recall**	-.41 ***;.46***; -.10	-.51 ***;.56***; .16

**Working Memory**	-.49 ***;.65****; -.19	-.11;.10; .09

**Motor function**	-.50 ***;.55***; .20	-.38 *;.42**; .16

*P < 0.05; **P < 0.005; ***P < 0.001; **** *P *< 0.0001. Age and education influenced neuropsychological performance, and this was more pronounced in the control group. Gender did not have a significant effect on neuropsychological performance.

### NP tests performance of AIDS patients and non-AIDS HIV+ individuals

**Figure 1:**
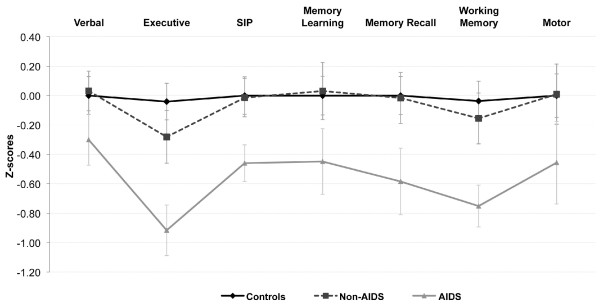
**Increased cognitive impairment in AIDS patients compared to non-AIDS and HIV-controls**. Mean (± standard error) ability z-scores for each group was computed for the seven ability domains, analyzed using ANOVA and post hoc Dunnett's comparison. Compared to controls and HIV+ non-AIDS individuals, z-scores of AIDS patients were significantly lower on Executive function, Speed of Information Processing, Memory Recall and Working memory (*p *< 0.01).

The AIDS subgroup of HIV+ volunteers performed significantly worse on the NP tests than either the HIV- controls (P = 0.005) or the non-AIDS HIV+ subgroup (P < 0.01). Compared to the mean z-score for the overall performance of HIV- controls (-0.01 ± 0.7), the mean z-score for the overall performance of the AIDS subgroup was -0.54 ± 0.63 (P = 0.005), while mean z-score of non-AIDS HIV+ subgroup was -0.05 ± 0.56. Adjusting performance for age and education yielded comparable results. The AIDS and non-AIDS HIV+ subgroups had the same demographic characteristics and when NP performance was adjusted for age and education, AIDS patients still performed worse than non-AIDS and controls. Analysis of mean ability z-scores of controls, AIDS, and non-AIDS for each of the 7 ability domains showed that AIDS patients performed significantly worse (P < 0.01) than controls in NP tests assessing executive function, speed of information processing, memory recall, and working memory (Figure 1). Analyses adjusting for demographic effects (age, education, gender) gave similar results. Compared to controls, individuals in the AIDS group also had lower mean z-scores on tests assessing verbal, memory learning, and motor functions, but the difference was not statistically significant. Similarly, NP performance of HIV- and non-AIDS HIV+ groups showed no significant difference in all 7 ability domains tested (Figure 1).

Computation of effect sizes when comparing the HIV- controls versus the AIDS subgroup, and HIV- versus the non-AIDS HIV+ subgroup, showed medium-to-large effect sizes for overall cognitive and executive function and working memory (Table 5). Effect sizes were medium for speed of information processing and memory recall, and small-to-medium for verbal fluency, learning, and motor function (Table 5). Global and ability domain mean z-score effect sizes showed no difference between the overall performance of non-AIDS HIV+ subgroup and HIV- controls (Tables 5), however a small effect of non-AIDS HIV infection was observed for executive function.

**Table 5:** Effect sizes between controls, Non-AIDS and AIDS for cognitive domains ability scores and global score

	Non-AIDS	AIDS	AIDS
Ability domain	Vs.	Vs.	Vs.
	Controls	Controls	Non-AIDS
**Mean Z-score**	0.06	0.74	0.78

**Verbal**	-0.04	0.34	0.44

**Executive function**	0.28	1.03	0.74

**Speed information processing**	0.02	0.57	0.71

**Memory Learning**	-0.03	0.46	0.47

**Memory Recall**	0.02	0.60	0.57

**Working Memory**	0.13	0.84	0.77

**Motor function**	-0.01	0.40	0.39

All effect sizes were corrected for small sample size bias using Hedges' correction.

## Discussion

This is the first study of NP performance and impairment of specific cognitive domains using a comprehensive and validated NP assessment battery in HIV-infected individuals in Cameroon. After translation into French and when administered by trained examiners, this battery detected cognitive dysfunction in HIV-infected Cameroonians. Participants were able to understand, follow test instructions, and validly complete NP tests, including computerized tests such as the Category test, WCST-64, and PASAT-50. All HIV- and HIV+ subjects were recruited in the same settings/hospitals in Yaoundé, Cameroon, and shared similar demographic features. As in many SSA countries, it has been shown that HIV transmission in Cameroon is predominantly (> 90%) through unprotected heterosexual contact [50] which reduces the likelihood of comorbid conditions that may affect the CNS (e.g., substance use disorders, co-infection with Hepatitis-C due to injection drug use that is a common risk for HIV infection in other parts of the world). Cultural differences probably affect performances on NP tests in a similar way as education differences. However this will not necessarily affect the validity of the NP tests for measuring disease related CNS dysfunction because the basic abilities assessed (verbal fluency, learning, attention and working memory, cognitive processing speed, executive function) exist and have some relevance in any population. Moreover, these cultural differences are mitigated when controls and diseased groups are from the same population with similar culture, as in this study.

HIV infection in Cameroon appears to be associated with impairment of cognitive function in domains similar to those most affected by HIV in geographic regions outside West Africa. Specifically, AIDS patients performed worse than non-AIDS HIV+ and HIV- groups on the global NP testing and NP tests assessing executive function, speed of information processing, working memory, and memory recall. In general, compared to HIV- control, the pattern of cognitive deficits was similar in non-AIDS HIV+ and AIDS groups, with deficits more pronounced in AIDS patients for all cognitive domains. Our results also indicate that neurocognitive impairment increases as the disease progress and is strongly associated with advanced immune deficiency and clinical disease, consistent with findings in the developed world. Effects from opportunistic infections are unlikely to have caused NP impairment because persons with a history of these diseases were excluded. All study participants were recruited at Yaoundé hospital out-patient clinics, appeared physically well and came to the clinics alone, without any support from a caregiver; thus, it is unlikely that patients in the AIDS group performed poorly because they were feeling too sick to perform at their best.

Other studies have shown increased rates of neurocognitive impairment with advanced stages of HIV infection [51-53]. Using this comprehensive NP battery, Heaton and colleagues [28,29] showed that HAND is an important feature of HIV infection in China, and the impairment pattern, severity, and prevalence were similar to those reported in Western countries. Studies in India showed deficits in the domains of fluency, working memory, memory and learning in Clade C HIV-infected individuals, with AIDS patients showing greater impairment in visual working memory than non-AIDS and seronegative controls [32]. Robertson and colleagues [54] evaluated NP performance among HIV+ individuals in Uganda and found that compared to HIV- controls, the HIV+ group showed significant deficits on measures of learning and memory, speed of information processing, attention and executive function.

Clifford and colleagues [55] examined a group of workers in Ethiopia and found that HIV+ patients had slowed finger-tapping speed, but that the HIV+ and HIV- groups performed similarly in timed gait, Grooved Pegboard, and verbal fluency tests. Similarly, we found that performance of HIV- and HIV+ groups on the Grooved Pegboard, and on tests of verbal fluency, and on Trail making-A or Color Trails-I and II did not differ significantly (Table 1). In contrast, our more comprehensive NP battery found that HIV+ persons (mainly those with advanced disease) performed significantly worse than HIV- controls on tests of working memory (PASAT and WMS-III Spatial Span) and higher level executive functions (Category Test and WCST-64), with smaller (non significant with the current sample sizes) effect sizes in the same direction on tests of speed of information processing (WAIS-III Digit Symbol and Symbol Search, and Stroop Color Naming speed) and visual episodic memory (BVMT-R). Interestingly, in contrast to the latter result with visual episodic memory and findings in developed countries, our measures of verbal episodic memory (HVLT-R learning and recall) did not even show a small HIV effect. A smaller HIV effect for our verbal than visual episodic memory measures also was seen in rural China [29] and may suggest that the visual measures are more generalizable across cultures.

Several studies had demonstrated that demographic effects such as age, education, cultural and ethnic differences affect NP test performance [56-59]. A study of NP impairment among HIV+ individuals in South Africa showed that older age, lower education level, post-traumatic stress disorder, and alcohol use affected NP performance [60]. As expected, demographic effects were also present in our data as computation of Pearson's r showed that age and education significantly influenced NP performance, especially in the HIV- group, while gender had no major effect.

The cognitive impairments observed among HIV+/AIDS Cameroonians are likely due to HIV infection because our comparison groups were well matched for demographic factors and those with confounding conditions such as alcohol intoxication, systemic illness, CNS or psychiatric diseases were excluded. There are currently no normative adult NP test data for Cameroon or SSA, and large demographic effects noted in this study underscore need for more comprehensive local normative NP data to improve neurocognitive diagnosis [61]. Our subsequent studies will enroll more HIV- control subjects in order to establish normative data for NP testing in adult Cameroonians.

Our pilot study has a number of limitations including: 1) the small sample size which likely limits the power to detect differences in some individual NP test measures, precludes us from creating demographically adjusted test scores and from exploring possible effects of ART on cognitive performance; 2) the inability of the current study to estimate the population prevalence of cognitive impairment due to lack of local normative NP data; 3) lack of brain imaging and cerebrospinal fluid (CSF) data, due to limited resources and logistic difficulties, precludes us from exploring the relationship between HIV-induced neurologic abnormalities, HIV brain infection (CSF viral load) and cognitive performance; 4) this study was conducted in an urban setting and findings cannot be generalized to rural settings where inhabitants have much lower education levels.

A recent United Nations Programme on HIV and AIDS (UNAIDS) report estimates that over half million Cameroonians are currently living with HIV, of which only about 25% have access to antiretroviral drugs and ART coverage for prevention of mother-to-child transmission is less than 25% [17]. In our current study, only 15 of the 44 HIV+ individuals (34%) were on ART, including 6 of the 22 non-AIDS HIV+ and 9 of the 22 AIDS patients. In subsequent studies with larger sample size, we will evaluate the effect of ART on neurocognitive performance. Neurocognitive impairment predicts the presence of HIV encephalitis [62-64] and is an independent risk factor for death [65]. In resource limited countries like Cameroon, many HIV-infected individuals have no access to ART, and low CD4 cell counts and clinical symptoms remain the major criteria for selecting patients for ART treatment. Detection of neurocognitive impairment could target patients for ART, possibly reversing brain dysfunction in some cases, improving their productivity and quality of life, and perhaps even prolonging their survival.

## Conclusions

Advanced immunosuppression from HIV infection is associated with HAND in Cameroon. AIDS patients showed impairment in executive function, speed of information processing, working memory, and memory recall and the pattern of deficits is similar to that found in many other areas of the world. Further longitudinal research with large HIV-infected population is needed to determine whether suppression of HIV replication through ART will reduce the prevalence of neurocognitive impairment in Cameroon as observed elsewhere.

## Abbreviations

AIDS: acquired immune deficiency syndrome; ART: antiretroviral therapy; CDC: center for disease control; HAND: HIV-associated neurocognitive disorders; HIV: human immunodeficiency virus; SSA: sub-Saharan Africa; CNS: central nervous system; IHDS: International HIV Dementia Scale; NP: neuropsychological; HNRC: HIV Neurobehavioral Research Center.

## Competing interests

The authors declare that they have no competing interests.

## Authors' contributions

GDK was responsible for the study conception and design, data analysis and writing of the paper. AKN contributed to study design, coordinated subject recruitment, data acquisition and edited the paper. LAC and RKH contributed to data quality assurance, analysis and interpretation, and edited the paper. KTC, JYF, SE, RD, and DMN contributed to subject recruitment, counseling, neuropsychological, neurological and general medical evaluation, and data acquisition. EN and DM performed laboratory testing and data acquisition; FB contributed to study design and subject recruitment. RJE and JAM contributed to study design and edited the paper. DRF contributed to the training of Cameroon investigators and data quality assurance. All authors have approved the final version of this manuscript for submission.

## Authors' information

**United States investigators**: GDK is Associate Professor and Vice-Chair in the Department of Pharmacology and Experimental Neurosciences, University of Nebraska Medical Center. RKH is Professor and Neuropsychologist at HNRC, University of California San Diego (UCSD). LAC was a Research Fellow at HNRC and recently moved to the University of New South Wales, Sydney, Australia. RJE is a Neurologist and Professor at the HNRC, UCSD. JAM is Professor, Epidemiologist, and infectious diseases specialist at the HNRC, UCSD. DRF is a Psychometrist at the HNRC, UCSD.

**Cameroon investigators**: AKN is a Neurologist, Head of the Neurology Department at the Yaoundé Central Hospital, and Associate Professor and Vice-Dean, Faculty of Medicine and Biomedical Sciences, University of Yaoundé I. KTC is a Neurologist at the Yaoundé Central Hospital. JYF is a clinician and research fellow at the Yaoundé Central Hospital. SE and RD are Psychologists and trained Psychometrists at the Yaoundé Central Hospital. DM is Head of the hematology lab at the Yaoundé University Hospital Center, and Professor in the Faculty of Medicine and Biomedical Sciences, University of Yaoundé I. EM is a technician in the hematology lab, University Hospital Center, Yaoundé. FB is Professor in the Faculty of Medicine and Biomedical Sciences, University of Yaoundé I. DMN is a counselor and social worker at the HIV Day-Care Hospital, Yaoundé Central Hospital.

## Pre-publication history

The pre-publication history for this paper can be accessed here:

http://www.biomedcentral.com/1471-2377/10/60/prepub
